# Murine Dishevelled 3 Functions in Redundant Pathways with Dishevelled 1 and 2 in Normal Cardiac Outflow Tract, Cochlea, and Neural Tube Development

**DOI:** 10.1371/journal.pgen.1000259

**Published:** 2008-11-14

**Authors:** S. Leah Etheridge, Saugata Ray, Shuangding Li, Natasha S. Hamblet, Nardos Lijam, Michael Tsang, Joy Greer, Natalie Kardos, Jianbo Wang, Daniel J. Sussman, Ping Chen, Anthony Wynshaw-Boris

**Affiliations:** 1 Department of Pediatrics, School of Medicine, University of California San Diego, La Jolla, California, United States of America; 2 Emory University School of Medicine, Atlanta, Georgia, United States of America; 3 Columbus State Community College, Columbus, Ohio, United States of America; 4 Department of Microbiology and Molecular Genetics, University of Pittsburgh School of Medicine, Pittsburgh, Pennsylvania, United States of America; 5 New Horizons Diagnostics, Columbia, Maryland, United States of America; 6 Department of Pediatrics and Institute for Human Genetics, University of California San Francisco School of Medicine, San Francisco, California, United States of America; Harvard Medical School, United States of America

## Abstract

Dishevelled (Dvl) proteins are important signaling components of both the canonical β-catenin/Wnt pathway, which controls cell proliferation and patterning, and the planar cell polarity (PCP) pathway, which coordinates cell polarity within a sheet of cells and also directs convergent extension cell (CE) movements that produce narrowing and elongation of the tissue. Three mammalian *Dvl* genes have been identified and the developmental roles of *Dvl1* and *Dvl2* were previously determined. Here, we identify the functions of *Dvl3* in development and provide evidence of functional redundancy among the three murine *Dvls. Dvl3* ^−/−^ mice died perinatally with cardiac outflow tract abnormalities, including double outlet right ventricle and persistent truncus arteriosis. These mutants also displayed a misorientated stereocilia in the organ of Corti, a phenotype that was enhanced with the additional loss of a single allele of the PCP component *Vangl2/Ltap* (*LtapLp*/+). Although neurulation appeared normal in both *Dvl3* ^−/−^ and *LtapLp*/+ mutants, *Dvl3* ^+/−^;*LtapLp*/+ combined mutants displayed incomplete neural tube closure. Importantly, we show that many of the roles of *Dvl3* are also shared by *Dvl1* and *Dvl2*. More severe phenotypes were observed in *Dvl3* mutants with the deficiency of another *Dvl*, and increasing *Dvl* dosage genetically with *Dvl* transgenes demonstrated the ability of *Dvls* to compensate for each other to enable normal development. Interestingly, global canonical Wnt signaling appeared largely unaffected in the double *Dvl* mutants, suggesting that low *Dvl* levels are sufficient for functional canonical Wnt signals. In summary, we demonstrate that *Dvl3* is required for cardiac outflow tract development and describe its importance in the PCP pathway during neurulation and cochlea development. Finally, we establish several developmental processes in which the three *Dvls* are functionally redundant.

## Author Summary

Multi-gene families, comprising a set of very similar genes with shared nucleotide sequences, are common in mammals. Individual family members may be expressed in different places and perform separate functions. Alternatively, the genes may have redundant functions, but distinct dosage requirements. Mammals share three *Dishevelled* (*Dvl*) family members and while the roles of *Dvl1* and *Dvl2* have been described previously, the functions of *Dvl3* have remained elusive. Here, we show that the lack of *Dvl3* in mice affects the formation of the heart, neural tube, and inner ear. We further show that the defects in these tissues are much more severe when the mice are deficient in more than one *Dvl* family member, indicating redundant functions for these genes. Congenital heart disease affects approximately 75 in every 1,000 live human births, and approximately 30% of these diseases are due to disruptions in the outflow tract, the region affected in mice lacking *Dvl* genes. Neural tube defects, similar to those observed in the *Dvl* mutants, are also common in humans. The animal models described here provide useful tools to elucidate the genetic mechanisms that underlie these abnormalities and may provide novel ways of treating these disorders in the future.

## Introduction

Normal mammalian development is the result of complex signaling networks that regulate and coordinate cell behavior. Wnt signaling controls a broad spectrum of cell fate decisions during embryogenesis and is critical for cell to cell communication in mammalian development. Through the activation of specific target genes, the canonical Wnt pathway tightly regulates cell proliferation, differentiation, adhesion and survival, and controls embryonic patterning [1],[2]. A non-canonical Wnt planar cell polarity (PCP) pathway, parallel to that first discovered in flies, has been described in mammals, where it regulates cell polarity and convergent extension (CE) movements. In these coordinated cell movements, cells migrate medially and intercalate, producing an elongation and narrowing of the tissue along the anterior-posterior axis [3]–[7].

Dishevelled (Dvl) proteins, of which three have been identified in humans and mice [8]–[13] are highly conserved components of both the canonical Wnt and PCP signaling cascades. They function as essential scaffold proteins that interact with diverse proteins, including kinases, phosphatases and adaptor proteins [14],[15]. In the canonical Wnt pathway, Dvl transduces the signal activated by Wnt binding to membrane-bound Frizzled (Fz) receptors and low-density lipoprotein-related receptor protein (LRP) 5/6 co-receptors, causing the stabilization and cytosolic accumulation of the critical mediator, β-catenin. Following the translocation of β-catenin to the nucleus, it then binds with members of the T-cell factor (TCF)/lymphocyte enhancer factor (Lef) family of transcription factors to regulate the expression of target genes. Dvl is also one of the core components of the PCP signaling pathway, in addition to Fz, Van Gogh/Strabismus (Vang/Stbm), Flamingo/Starry night (Fmi/Stan), Diego (Dgo) and Prickle (Pk). The specific, highly controlled, asymmetric arrangement of these proteins allows polarity of the cell to be established within the plane of the epithelium and promotes the rearrangement of the cytoskeletal components of the cell.

In the heart, normal development of the cardiac outflow tract requires the addition of cells from the secondary heart field (SHF) dorsal to the primary heart tube [16],[17]. SHF cells, which express the LIM-homeodomain transcription factor *Islet1* (*Isl1*), migrate from the pharyngeal mesoderm to contribute to the myocardium of the outflow tract [18]. Recently canonical Wnt signaling has been shown to play a major role in the proliferation and expansion of SHF cells [19]–[22]. It has further been demonstrated that the canonical Wnt signaling mediator β-catenin directly activates *Isl1* expression [23]. Extensive remodeling of the outflow tract region with the addition of cardiac neural crest (CNC), mesenchyme from the crest of the neural folds, is then required to septate the single vessel to form the aorta and pulmonary artery [24]. Connections must form between the left ventricle and the aorta, as well as the right ventricle and pulmonary artery, to successfully establish both systemic and pulmonary circulation. Defects in the development of the outflow tract region cause phenotypes such as double outlet right ventricle (DORV), where both the pulmonary artery and the aorta connect to the right ventricle, transposition of the great arteries (TGA) and persistent truncus arteriosis (PTA), where the outflow tract fails to divide into an aorta and pulmonary artery.

Studies of *Looptail (LtapLp)* mice, which carry a missense mutation in *Van Gogh-like 2* (*Vangl2*, *strabismus*) [4],[25], a component of the PCP pathway, have also implicated non-canonical Wnt signaling mechanisms in mammalian heart development. *LtapLp/LtapLp* homozygous mutants display the outflow tract abnormality DORV [26]. Reports indicate that the PCP pathway, which is necessary for the polarized migration of myocardial cells to the outflow tract septum, is disrupted in these mutants [27],[28]. Similar outflow tract defects including TGA and PTA have also been observed in mice lacking the non-canonical signals *Wnt11* [29] and *Wnt5a* [30]. *Wnt5a* has been proposed to function in the outflow tract by stimulating intracellular increases in Ca^2+^ to regulate cells of the CNC.

PCP signaling is also required for the normal development of the auditory sensory organ, the organ of Corti [3],[5],[6],[31]. This structure is comprised of one row of inner and three rows of outer sensory hair cells, interdigitated with supporting cells such as inner phalangeal cells, the inner and outer pillar cells and the Deiters cells (reviewed in [32]). The sensory hair cells have stereociliary bundles uniformly orientated on the apical surfaces, which is the most obvious example of planar cell polarity in mammals. Consistent with this, mouse mutants of core PCP components including *Vangl2/Ltap* [5], *Fz*3 and *Fz6* [33],[34] and *Dvl1* and *Dvl2* [6] display stereocilia misorientation, indicating that the correct alignment of these stereocilia is dependent on functional PCP signaling [32]. Cellular rearrangements characteristic of CE movements are also required during the development of the organ of Corti [6],[35], during which a thicker and shorter postmitotic primordium undergoes integrated cellular intercalation movements to produce extension along the longitudinal axis and narrowing along a perpendicular axis. Therefore signaling via the PCP pathway is responsible for both the polarized extension and the establishment of planar cell polarity in the organ of Corti.

The neural plate undergoes narrowing and lengthening attributable to CE movements during mammalian neurulation. When PCP signaling is disrupted, cells of the neuroepithelium fail to intercalate, preventing the neural tube from fusing at the midline [36]–[39]. Severe neural tube closure defects are observed in mice carrying mutations in PCP components including *Vangl2/Ltap* [4],[25], *Dvl1* and *Dvl2* [6],[40], *Celsr* (a homolog of *flamingo*) [3] and *Fz*3 and *Fz6* [34]. These mutants have shorter and wider neural plates and display craniorachischisis, a completely open neural tube from mid-brain to tail.

To elucidate the role of specific *Dvls* in mammalian development, we have generated mouse knockouts for each of the *Dvl* genes. Interestingly distinct phenotypes were revealed, suggesting separate functions for the Dvl proteins. *Dvl1* knockout mice are viable and fertile, but display social interaction abnormalities and defects in sensorimotor gating [41]. By contrast, incompletely penetrant cardiac outflow tract abnormalities (50%) and rib/vertebral malformations (>90%) are observed in *Dvl2* knockout mice [40]. However, functional redundancy among the *Dvl* genes is also suggested from their overlapping expression patterns, as well as their high degree of conservation. In support of this, we have previously shown that *Dvl1* ^−/−^;*Dvl2* ^−/−^ mutants display craniorachischisis, a completely open neural tube, and abnormalities in the organ of Corti, both novel phenotypes not observed in the single *Dvl* knockouts [38],[40].

Here we describe the phenotype of mice deficient in *Dvl3* and determine its importance in conotruncal, cochlear and neural tube development. Given the overlapping expression patterns of the Dvls, as well as their high degree of conservation, we further addressed the possibility of functional redundancy and demonstrate that many of the roles of *Dvl3* are also shared by *Dvl1* and *Dvl2*. We attribute several of the developmental functions of *Dvl3* to its role in PCP signaling, enhancing our knowledge of this essential pathway in mammalian development and further defining the specific role of individual *Dvl* genes.

## Results

### Cardiac Defects in *Dvl3* ^−/−^ Mutants

**Figure 1:**
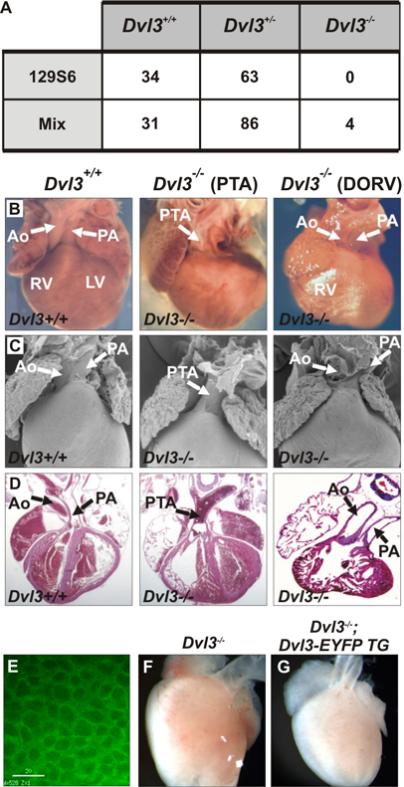
Cardiac defects of *Dvl3* knockout mice. The genotypes of weaning age pups from *Dvl3* heterozygote crosses, in both an inbred 129S6 and mixed genetic background, were collected in order to calculate the frequency of survival of *Dvl3* ^−/−^ mice (A). Cardiac abnormalities were observed in all *Dvl3* ^−/−^ mutants examined in an inbred background (B–D). Whole mounts (B), scanning electron microscopy images (C) and hematoxylin and eosin stained sections (D) of P0 hearts of *Dvl3* ^+/+^ (left) and *Dvl3* ^−/−^ hearts that displayed PTA (middle) and DORV (right). Aorta (Ao), pulmonary artery (PA), right ventricle (RV), left ventricle (LV). Deconvolution microscopy image showing EYFP-Dvl3 BAC transgene expression in the heart at E9.5. White bar = 20 µm (E). Addition of the *Dvl3-EYFP* transgene (G) rescues the abnormal cardiac phenotype of *Dvl3* ^−/−^ mutants (F, heart displays PTA).

*Dvl3* knock out mice were successfully generated (Figure S1). In order to calculate the frequency of survival of *Dvl3* ^−/−^ mice, the genotypes of pups from *Dvl3* heterozygote crosses were analyzed at weaning age (Figure 1A). In a 129S6 inbred background, 97 pups were genotyped. No *Dvl3* ^−/−^ pups survived to weaning, while *Dvl3* ^+/+^ and *Dvl3* ^+/−^ pups were observed in an approximate 1∶2 ratio (34 and 63 respectively), as expected from Mendelian ratios. In a mixed genetic background, 121 pups were analyzed, but only 4 out of the expected 30 *Dvl3* ^−/−^ mice (13.3%) survived. In contrast, at E18.5 *Dvl3* ^+/+^, *Dvl3* ^+/−^ and *Dvl3* ^−/−^ genotypes were recovered in normal Mendelian ratios from heterozygous crosses. These data indicate that 100% of *Dvl3* homozygotes in a 129S6 inbred background and approximately 87% in a mixed genetic background die shortly after birth. No gross abnormalities were observed in the few adult *Dvl3* ^−/−^ mice that did survive in a mixed background.

*Dvl3* ^−/−^ newborn pups had difficulty breathing and were often cyanotic. Examination of hearts from *Dvl3* ^−/−^ mutants at P0 (Figure 1B) using scanning electron microscopy (Figure 1C) and histochemical analysis (Figure 1D) revealed that all had conotruncal abnormalities. More specifically, seven of the eleven mutant hearts displayed PTA, the outflow tract having failed to divide into an aorta and pulmonary artery, and four showed DORV, where both the pulmonary artery and the aorta connected to the right ventricle.

We generated an EYFP-tagged *Dvl3* transgene using homologous recombination of BACs (Figure S2) and observed expression of Dvl3-EYFP in the embryonic heart during conotruncal development at E9.5 (Figure 1E). This transgene fully rescued the lethal defect in *Dvl3* ^−/−^ mutants, providing formal proof that the phenotype of the *Dvl3* ^−/−^ mutants is specifically due to the loss of *Dvl3*. From a cross between a *Dvl3* ^−/−^ rescued with the *Dvl3* transgene and a *Dvl3* heterozygote (both in a mixed background), 31% (37/118) of pups genotyped at weaning age were *Dvl3* ^−/−^ rescued with the transgene, whereas only 1.6% (2/118) *Dvl3* ^−/−^ mutants survived without the transgene. E18.5 hearts collected from *Dvl3* ^−/−^ embryos with the *Dvl3* transgene appeared normal and displayed no conotruncal abnormalities (Figure 1F and 1G).

### Conotruncal Defects in *Dvl3* ^−/−^ Mice Are Not Due to an Absence of CNC or SHF Cells

**Figure 2:**
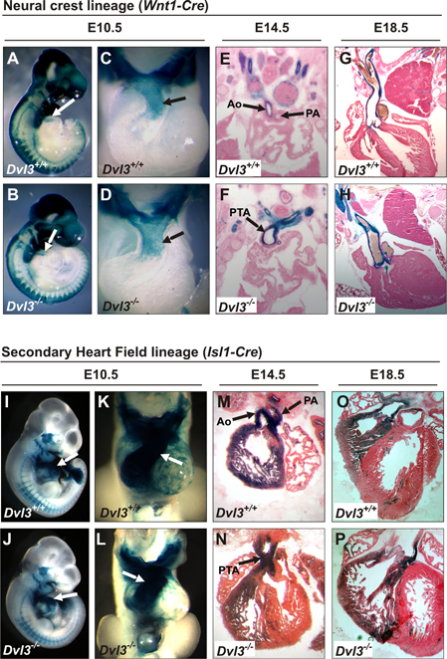
Cells of both the CNC and SHF lineage remain present in *Dvl3* ^−/−^ mutants. Lineage tracing experiments using lineage-specific Cre/LoxP recombination and a Rosa-26-lacZ Cre reporter that drives expression of β-galactosidase were performed to identify cells of the CNC and SHF in *Dvl3* ^−/−^ mutants. *Wnt1Cre* was used to specifically label cells from the neural crest (A–H) and *Isl1Cre* to label the SHF (I–P) at various stages of development in *Dvl3* ^+/+^ (A,C,E,G,I,K,M,O) and *Dvl3* ^−/−^ mutants (B,D,F,H,J,L,N,P). At E10.5 whole embryos (A,B,I,J) were dissected to visualize the conotruncus (C,D,K,L, arrows). At E14.5 (E,F,M,N) and E18.5 (G,H,O,P) hearts were sectioned to examine staining. Aorta (Ao), pulmonary artery (PA), persistent truncus arteriosis (PTA).

Normal development of the outflow tract requires contribution from both the CNC and SHF, so we examined whether a lack of either of these tissues in *Dvl3* ^−/−^ mutants may be responsible for the observed conotruncal defects by lineage tracing experiments using lineage-specific Cre/LoxP recombination and a *Rosa-26-lacZ Cre* reporter that expresses β-galactosidase only in cells with Cre activity. *Wnt1-Cre*, expressed in neural crest cells, was used to label the CNC cell population (Figure 2A–H) and SHF cells were specifically labeled using *Isl1-Cre* (Figure 2I–P). Embryos were collected and stained at E10.5, E14.5 and E18.5. At each of these stages both CNC and SHF cells were clearly evident in *Dvl3* ^−/−^ mutants, suggesting that the outflow tract defects were not due to an appreciable loss of either of these lineages.

### Neural Tube Defects in *Dvl3*;*LtapLp* Mutant Mice

**Figure 3:**
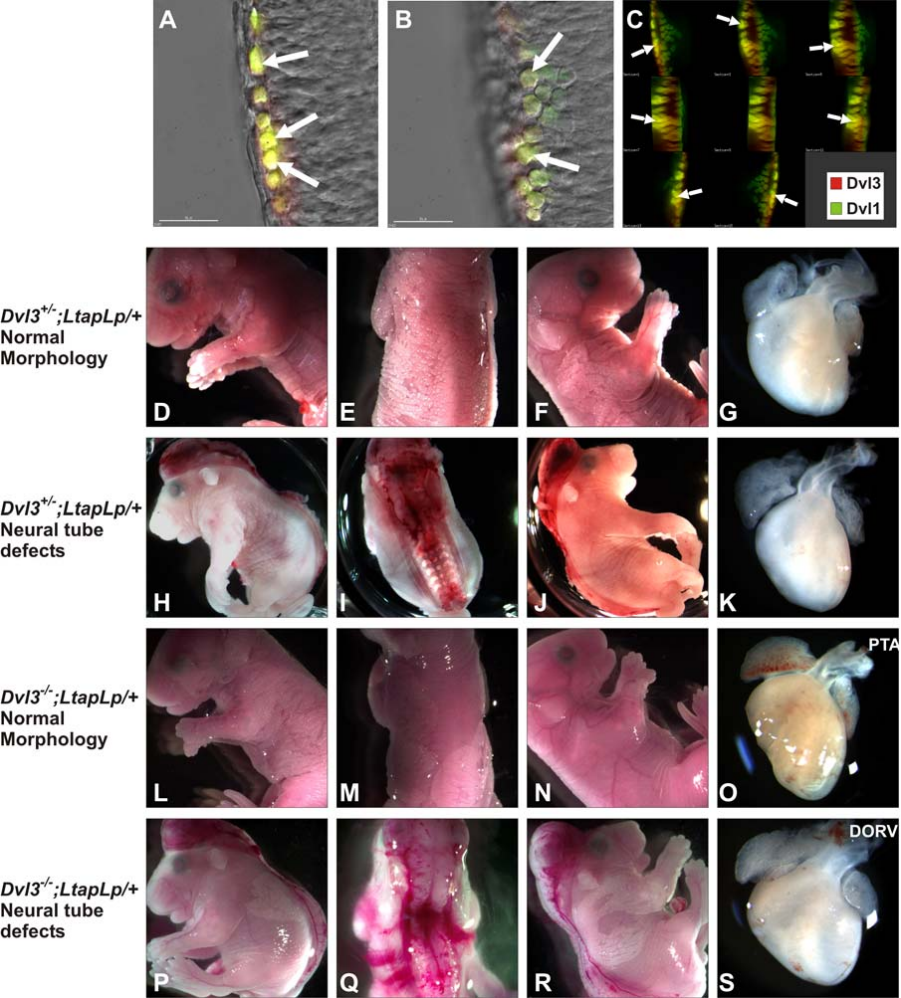
Neural tube defects in *Dvl3/LtapLp* mutants. Expression of Dvl1-ECFP transgene (green) and Dvl3-EYFP transgene (red) in the developing neural tube at E9.5 (A,B, both individual z-plane confocal images and C, a rotated z-stacked image). Colocalization of these Dvls was evident in many cells (white arrows). Several *Dvl3* ^+/−^;*LtapLp*/+ embryos collected at E18.5 appeared normal (D–F), but others displayed neural tube defects such as exencephaly or craniorachischisis (H–J). E18.5 *Dvl3* ^−/−^;*LtapLp*/+ embryos also showed both normal (L–N) and defective (P–R) neurulation. The hearts of these mutants were also examined (G,K,O,S) and appeared normal in *Dvl3* ^+/−^;*LtapLp*/+ embryos but conotruncal defects were displayed in *Dvl3* ^−/−^;*LtapLp*/+ mutants.

Neural tube defects were observed in *Dvl2* ^−/−^ mutants and to a greater extent in *Dvl1* ^−/−^;*2* ^−/−^ double knockouts, indicating functional redundancy [40]. We examined a potential role for *Dvl3* in neural tube closure. To determine expression of Dvl3 and Dvl1 in the developing neural tube we used transgenic mice carrying the *EYFP-Dvl3* transgene and another, similarly made *ECFP-Dvl1* transgene, which rescues the *Dvl1* ^−/−^;*Dvl2* ^−/−^ lethal phenotype (data not shown). Expression of both Dvl3 and Dvl1 was observed in the developing neural tube at E9.5 (Figure 3A–C), an important time for this developmental process. The colors were artificially changed to red and green for Dvl3 and Dvl1, respectively, for easy observation of colocalization (yellow). These Dvls appeared to colocalize in many cells (indicated with white arrows), but not in all cells.

Homozygous *LtapLp*;*LtapLp* mutants of the PCP signaling pathway component *Vangl2/Ltap*, display craniorachischisis, a completely open neural tube from mid-brain to tail [4],[25], whereas no neural tube defects are observed in *LtapLp*/+ heterozygotes. Although no neural tube defects were observed in any of the *Dvl3* ^−/−^ mutant embryos collected (data not shown), defective neurulation was apparent when we crossed *Dvl3* mutants with *LtapLp*/+ mice. Several *Dvl3* ^+/−^;*LtapLp*/+ embryos displayed normal neural tube development (Figure 3D–F), whereas others exhibited neural tube abnormalities such as craniorachischisis (Figure 3H–J) and exencephaly, defective closure of the rostral neural tube. Similar phenotypes were observed in *Dvl3* ^−/−^;*LtapLp*/+ mutants, with some appearing normal (Figure 3L–N) and others displaying craniorachischisis (Figure 3P–R). The frequency of these defects was similar in both *Dvl3* ^+/−^;*LtapLp*/+ (7/22, 32%, 5 with craniorachischisis and 2 with exencephaly) and *Dvl3* ^−/−^;*LtapLp*/+ mutants (6/16, 38%, all with craniorachischisis). However, craniorachischisis appears to be a more severe phenotype than exencephaly, indicating a more severe phenotype in *Dvl3* ^−/−^;*LtapLp*/+ mutants compared to *Dvl3* ^+/−^;*LtapLp*/+ mutants.

As both *Dvl3* ^−/−^ and *LtapLp*;*LtapLp* mutants display cardiac defects but the single heterozygotes do not, we looked for defects in the hearts of the *Dvl3* ^+/−^;*LtapLp*/+ double heterozygotes (Figure 3G and 3K). However, all hearts appeared normal, even in the mutants with neural tube defects, while all hearts from *Dvl3* ^−/−^;*LtapLp*/+ double mutant mice (Figure 3O and 3S) displayed conotruncal defects, as expected for *Dvl3* ^−/−^mice.

### Cochlea Defects in *Dvl3* ^−/−^ Mice

**Figure 4:**
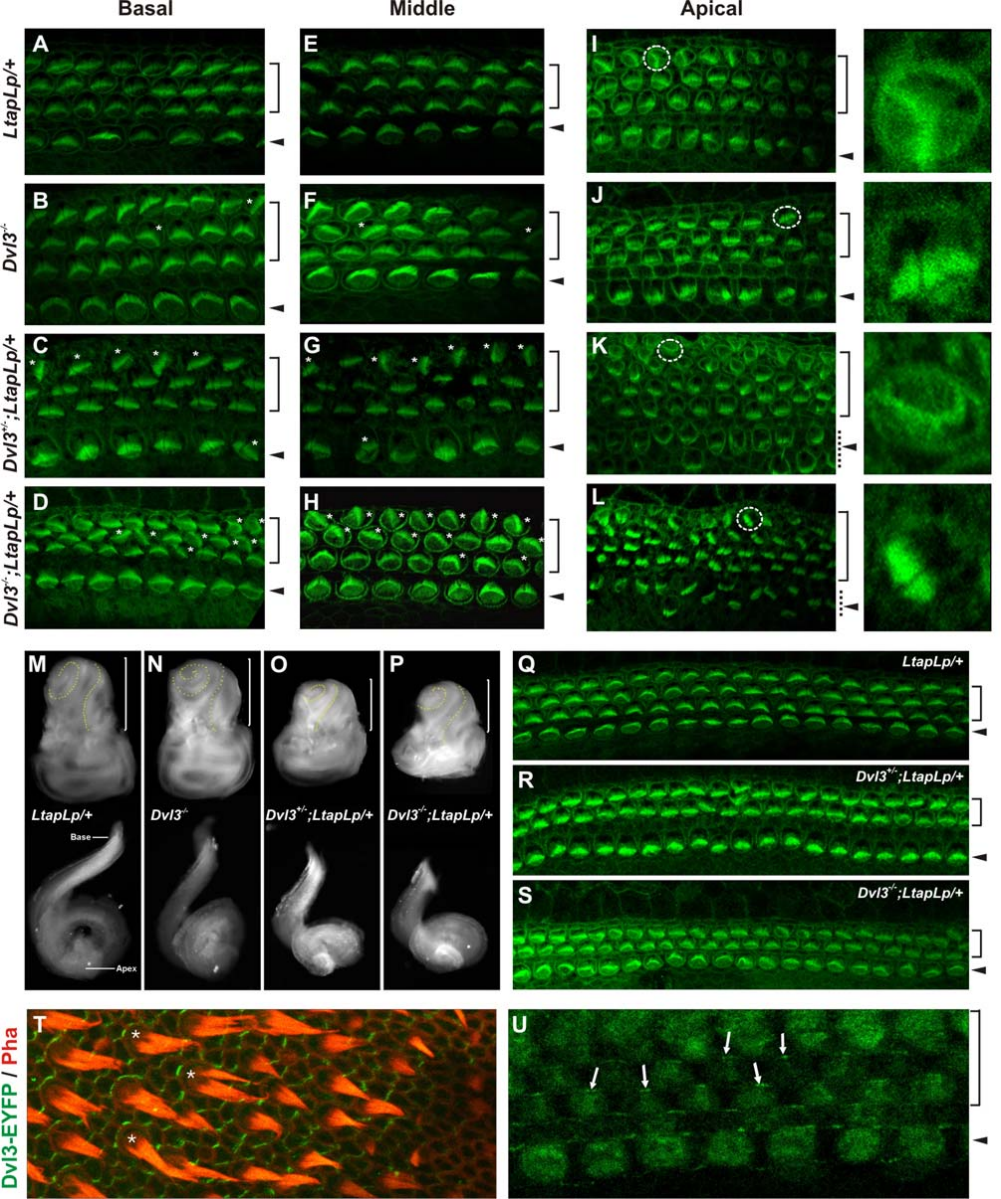
Cochlea defects in *Dvl3* ^−/−^ *and Dvl3/LtapLp* mutants. (A–L) Confocal images (100×) of the surface of cochlear whole mounts isolated at E18.5 from *LtapLp*/+ (A,E,I), *Dvl3* ^−/−^ (B, F,J), *Dvl3* ^+/−^;*LtapLp*/+ (C,G,K), *Dvl3* ^−/−^;*LtapLp*/+ (D,H,L). Images show comparable regions in each mouse at the base (A–D), middle (E–H) and apex (I–L) of the cochlear ducts. In A–L brackets and arrowheads indicate the outer and inner hair cells, respectively. In A–H white stars indicate misoriented stereociliary bundles and in I–L dotted circles in the left panels outline regions presented in the right panels. (M–P) Light micrographs of the inner ears (top) and isolated cochlea ducts (bottom) isolated at E18.5 from *LtapLp*/+ (M), *Dvl3* ^−/−^ (N), *Dvl3* ^+/−^;*LtapLp*/+ (O), *Dvl3* ^−/−^;*LtapLp*/+ (P) samples. The yellow dotted line traces each cochlear spiral and the brackets indicate the cochlea portion of the inner ear. (Q–S) Confocal images (40×) of the surface of cochlear whole mounts isolated at E18.5 from *LtapLp*/+ (Q), *Dvl3* ^+/−^;*LtapLp*/+ (R), *Dvl3* ^−/−^;*LtapLp*/+ (S) mutants. Images show comparable regions in each cochlea. (T) Expression of Dvl3-EYFP transgene (green) and stereocilary bundles (red) in the vestibular utricle region of the inner ear. White stars indicate cells in which the asymmetric localization of Dvl3 is very clear. (U) Expression of Dvl3-EYFP transgene in the cochlear duct. Arrows indicate the polarized localization of Dvl3, towards the lateral side of the sensory hair cells.

A major role for the mammalian PCP pathway in the development of the organ of Corti has previously been described. Cochleae from mouse mutants homozygous for a mutant allele of the core PCP component *Vangl2/Ltap* (*LtapLp*;*LtapLp*), display misorientation of stereociliary bundles in sensory hair cells due to disrupted planar cell polarity and the cochlear ducts are also shortened and wider due to defects in CE cell movements [5],[6]. We have shown that both Dvl1 and Dvl2 function in PCP signaling in the developing cochlea [6]. To assess whether Dvl3 also functions in this process, we examined cochleae from *Dvl3* mutant embryos, with and without an extra *LtapLp* mutation in *Vangl2*. No cochlear defects were observed in either *Dvl3* ^+/−^ (data not shown) or *LtapLp*/+ single heterozygotes in the basal and middle regions (Figs 4A and 4E). However, a mild PCP phenotype was observed in *Dvl3* ^−/−^ cochleae, where the uniform orientation of stereociliary bundles was disrupted in some hair cells in both the base and the middle of the cochlear ducts (Figure 4B and 4F, respectively). The cochleae of *Dvl3* ^+/−^;*LtapLp*/+ mutants that had normal neural tube development were unaffected. However, in the *Dvl3* ^+/−^;*LtapLp*/+ mutants that showed defective neurulation, a PCP phenotype in the cochlea accompanied the neural tube defect, with the misorientation of several sensory hair cells in both the basal and middle regions (Figure 4C and 4G, respectively). Compared to these two mutants the severity of the phenotype was much increased in *Dvl3* ^−/−^;*LtapLp*/+ mutants that showed neural tube defects, in which many hair cells had disrupted orientation (Figure 4D and 4H). Misorientation of stereociliary bundles was also observed in *Dvl3* ^−/−^;*LtapLp*/+ mutants that had normal neural tube development, but the phenotype was much weaker than in embryos of the same genotype displaying defective neurulation. In the apical regions of the cochlear ducts, rotated stereociliary bundles were observed in the hair cells of *LtapLp*/+ (Figure 4I), *Dvl3* ^−/−^ (Figure 4J), *Dvl3* ^+/−^;*LtapLp*/+ (Figure 4K) and *Dvl3* ^−/−^;*LtapLp*/+ (Figure 4L) mutants, although the number of cells affected was increased in both *Dvl3* ^+/−^;*LtapLp*/+ and *Dvl3* ^−/−^;*LtapLp*/+ mutants. In these combined mutants the degree of rotation also appeared more severe, as several stereociliary bundles were completely reversed (Figure 4K).

In addition to rotated stereociliary bundles in the sensory hair cells, strong patterning defects were also observed in *Dvl3*;*LtapLp* mutant cochleae. Separately, the patterning in the cochleae of *Dvl3* ^−/−^ and *LtapLp*/+ single mutants appeared normal (Figure 4M and 4N). However, the inner ears of *Dvl3* ^+/−^;*LtapLp*/+ and *Dvl3* ^−/−^;*LtapLp*/+ mutants with neural tube defects, were much smaller than in littermate controls (Figure 4O and 4P, respectively). Upon further dissection, the cochleae of the *Dvl3* ^+/−^;*LtapLp*/+ and *Dvl3* ^−/−^;*LtapLp*/+ mutants with craniorachischisis were also much shorter compared to controls (Figure 4M–P). At the cellular level, often in these *Dvl3* ^+/−^;*LtapLp*/+ and *Dvl3* ^−/−^;*LtapLp*/+ mutants the normal arrangement of 3 outer hair cell rows and 1 inner hair cell row was disrupted. In the apical region of *Dvl3* ^+/−^;*LtapLp*/+ and *Dvl3* ^−/−^;*LtapLp*/+ cochleae, additional rows of both outer and inner hair cells were observed (Figure 4K and 4L). Loss of outer hair cell rows was also detected in these mutants, so that the normal three rows became only two rows (Figure 4R and 4S). In total, this patterning defect was observed in 5–20% of the length of the cochlea.

Finally, the location of Dvl3 expression was determined in the inner ear sensory organs, including both the vestibular (Figure 4T) and cochlear (Figure 4U) end organs, using the EYFP-tagged *Dvl3* transgene. Dvl3-EYFP signals were asymmetrically localized in cells in all inner ear sensory regions. In the cochlea, Dvl3 was localized either on the lateral sides of the sensory hair cells, or on the medial side of the surrounding supporting cells, similar to what was observed for Dvl2 [6].

### No Skeletal Defects Were Observed in *Dvl3* ^−/−^ Mutants

We have previously reported both vertebral and rib malformations in *Dvl2* ^−/−^mutants and more severe skeletal defects in *Dvl1* ^−/−^;*Dvl2* ^−/−^ double knockouts [40]. Given the overlapping expression patterns and high homology between the *Dvls*, we also examined the skeletons of *Dvl3* ^−/−^ mutants. However, no rib or vertebral abnormalities were observed (data not shown). 7/11 *Dvl3* ^−/−^ mutants showed xiphoid bifurication, but this was also observed in several wild type controls.

### Phenotypes of *Dvl1*;*Dvl3* and *Dvl2*;*Dvl3* Double Mutants

**Figure 5:**
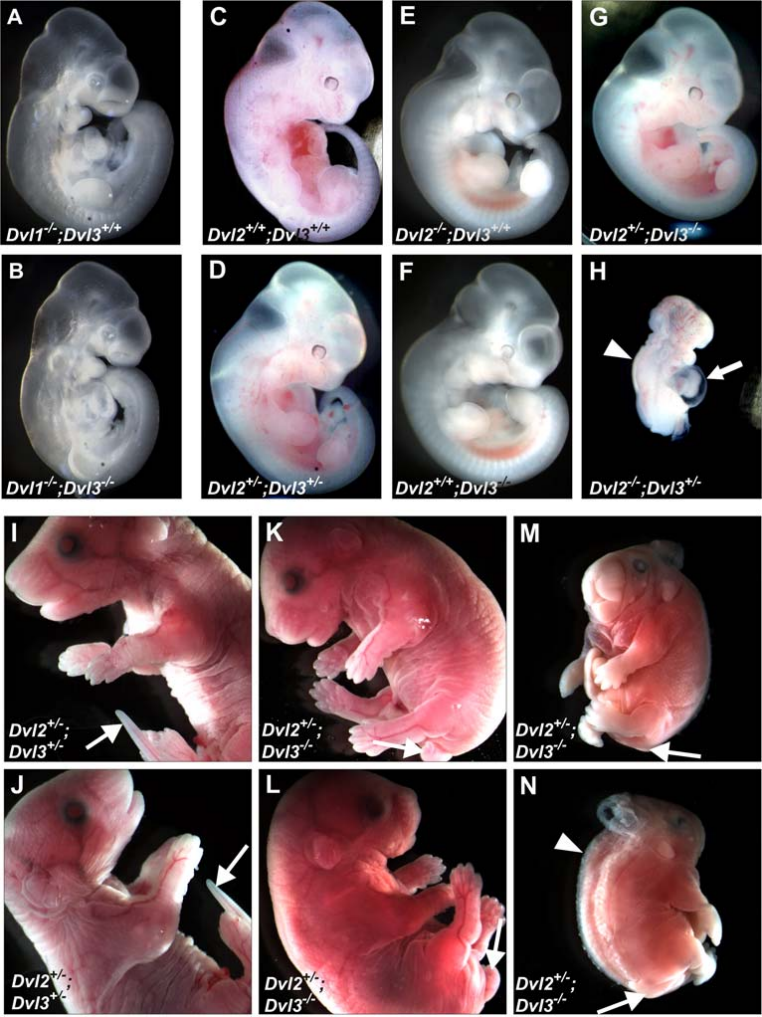
Phenotypes of double *Dvl* mutants at mid-gestation and late embryonic stages. Normal development was observed in *Dvl1* ^−/−^ (A) and *Dvl1* ^−/−^;*Dvl3* ^−/−^ mutants (B) at E10.5 (A,B), although these mutants die shortly after this stage, often between E13.5–15.5. Phenotypes of *Dvl2*;*Dvl3* double mutants collected at E11.5 (C–H). *Dvl2* ^+/−^;*Dvl3* ^+/−^ double heterozygotes (D) and *Dvl2* ^+/−^;*Dvl3* ^−/−^ mutants (G) appeared normal at this stage, but *Dvl2* ^−/−^;*Dvl3* ^+/−^ mutants displayed craniorachishisis, severely abnormal hearts and posterior truncation and die at approximately E9.5 (H). Phenotypes of varying severity were observed in *Dvl2* ^+/−^;*Dvl3* ^−/−^ mutants at E18.5 (I–N). Most appeared shortened in the A–P axis, with abnormal head shape, shortened snout, and a kinked tail (K,L). Two mutants also displayed craniorachischisis and more severe A–P truncation and one of these further showed gastroschisis and absence of tail (M,N).

Redundancy between *Dvl1* and *Dvl2* was evident from our studies of *Dvl1* ^−/−^;*Dvl2* ^−/−^ double mutants, which displayed novel phenotypes not observed in the single *Dvl* knockouts [38],[40]. To address possible redundancy between *Dvl3* and the other two *Dvls* we examined the phenotypes of *Dvl1*;*Dvl3* and *Dvl2*;*Dvl3* double mutants. Both *Dvl1* ^+/−^;*Dvl3* ^+/−^ and *Dvl1* ^−/−^;*Dvl3* ^+/−^ mutants survived to adulthood and were fertile. Normal development was also observed in *Dvl1* ^−/−^;*Dvl3* ^−/−^ embryos until E12.5 (Figure 5A and 5B), but these mutants died soon after of unknown causes, normally between E13.5 and E15.5. Importantly, no neural tube defects were observed.

At mid-gestation *Dvl2* ^+/−^;*Dvl3* ^+/−^ and *Dvl2* ^+/−^;*Dvl3* ^−/−^ double mutants also appear normal (Figure 5D and G), however a striking phenotype was observed in *Dvl2* ^−/−^;*Dvl3* ^+/−^ mutants (Figure 5H). These mutants do not appear to survive beyond E9.5 and display craniorachischisis, pericardial effusion and abnormal looping of the heart, as well as severe posterior truncation. No *Dv2* ^−/−^;*Dvl3* ^−/−^ embryos have been recovered from litters collected from E8.5 onwards, suggesting lethality earlier in development.

Defects are observed in *Dvl2* ^+/−^;*Dvl3* ^−/−^ double mutants at later developmental stages. At E18.5 these mutants appear shorter along the anterior-posterior (A–P) axis, show an abnormal head shape and a truncated snout, as well as a shortened and kinked tail (Figure 5K and L). Craniorachischisis was also observed in two *Dvl2* ^+/−^;*Dvl3* ^−/−^ mutants. One of these with the neural tube defect also showed several other severe phenotypes, including gastroschisis and absence of tail (Figure 5M and 5N).

### Cardiac Defects in *Dvl2*;*Dvl3* Double Mutants

**Figure 6:**
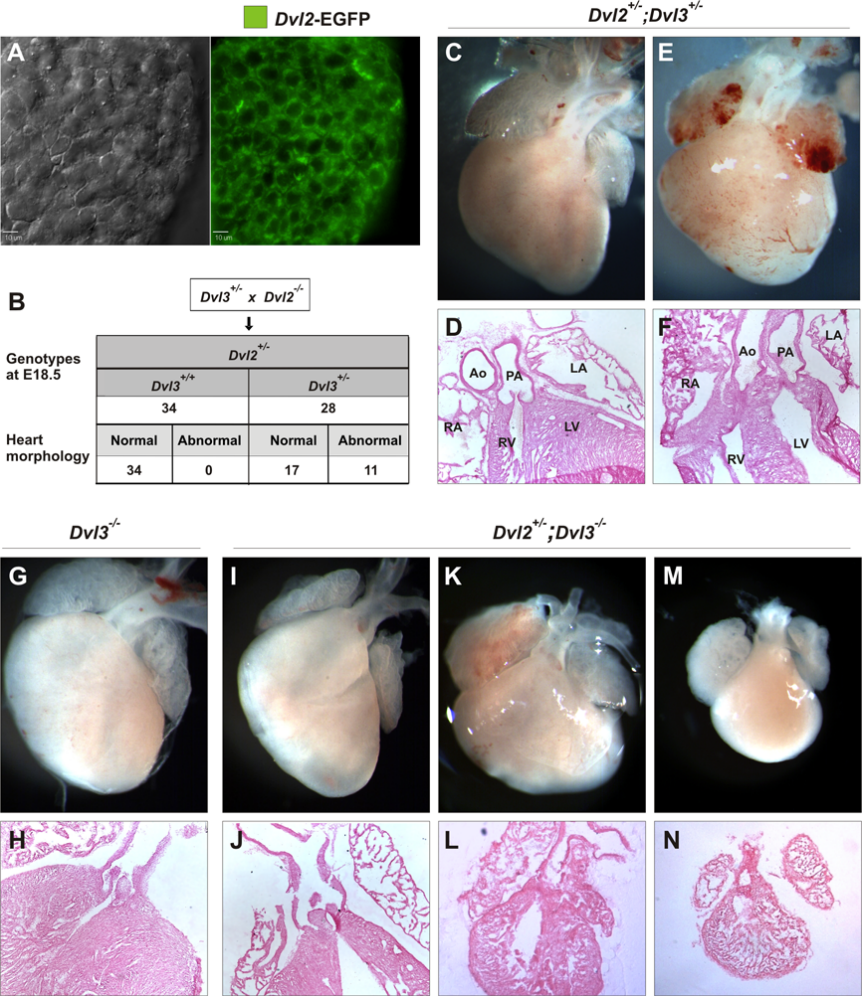
Cardiac abnormalities in *Dvl2* ^+/^^−^;*Dvl3* ^+/−^ and *Dvl2* ^+/^−^^;*Dvl3* ^−/−^ mutants. Deconvolution microscopy image showing EGFP-Dvl2 BAC transgene expression in the heart at E9.5 (A). The hearts of E18.5 embryos collected from *Dvl2* ^−/−^×*Dvl3* ^+/−^ crosses were examined and conotruncal abnormalities were observed in 11/28 *Dvl2* ^+/−^;*Dvl3* ^+/−^ hearts (B). Whole mount (C,E) and sectioned (D,F) E18.5 *Dvl2* ^+/−^;*Dvl3* ^+/−^ hearts showing normal morphology (C,D), DORV (E) and TGA (F). Whole mount (G, I, K, M) and sectioned (H, J, L, N) E18.5 *Dvl2* ^+/−^;*Dvl3* ^−/−^ hearts showing cardiac abnormalities with varying degrees of severity. Three (I,J) appeared similar to *Dvl3* ^−/−^ mutants (G,H), two had a ‘medium’ phenotype, which was worse than *Dvl3* ^−/−^ mutants (K,L) and two had a much worsened phenotype compared to *Dvl3* ^−/−^ mutants with severely altered morphology (M,N).

Since both *Dvl2* ^−/−^ and *Dvl3* ^−/−^ mutants both display conotruncal defects, we determined whether these *Dvls* have redundant or distinct functions in heart development. As common spatial and temporal expression patterns may indicate similar functions, we first confirmed that *Dvl3* (Figure 1E) and *Dvl2* (Figure 6A) shared comparable expression patterns in the heart at E9.5 during conotruncal development.

We first examined the hearts of *Dvl2* ^+/−^;*Dvl3* ^+/−^ double heterozygotes to see whether these mutants had similar defects as the homozygotes, which would suggest redundant functions for *Dvl2* and *Dvl3*. We observed that *Dvl2* ^+/−^;*Dvl3* ^+/−^ mice often survive to adulthood and are fertile. However, in an inbred background, conotruncal abnormalities were seen in 11/28 *Dvl2* ^+/−^;*Dvl3* ^+/−^ hearts examined at E18.5 (Figure 6B). These defects included 9 DORV, 1 PTA and 1 TGA (Figure 6C–F).

The hearts of *Dvl2* ^+/−^;*Dvl3* ^−/−^ mutants were also examined to determine whether an extra loss of one copy of *Dvl2* would worsen the phenotype of *Dvl3* ^−/−^ mutants. Three *Dvl2* ^+/−^;*Dvl3* ^−/−^ hearts collected at P0 (Figure 6I and 6J) had similar morphology to *Dvl3* ^−/−^ hearts (Figure 6G and 6H), 1 with DORV and 2 with PTA. However, four *Dvl2* ^+/−^;*Dvl3* ^−/−^ hearts collected appeared to have a worse phenotype than *Dvl3* ^−/−^ mutants with varying degrees of severity and all displaying PTA (Figure 6K–N). Two were slightly smaller and had lost the characteristic fist shape heart morphology, becoming less tapered towards the bottom (Figure 6K and 6L). The other two *Dvl2* ^+/−^;*Dvl3* ^−/−^ hearts were much reduced in size compared to *Dvl3* ^−/−^ mutants and had severely altered morphology, with a teardrop shape (Figure 6M and 6N). The embryos that the hearts were collected from were all alive, apart from the two severest phenotypes (Figure 6M and 6N), which were dead. After sectioning, it appeared that both right and left ventricles were still present in all samples.

### Functional Redundancy between Dvl Proteins

**Figure 7:**
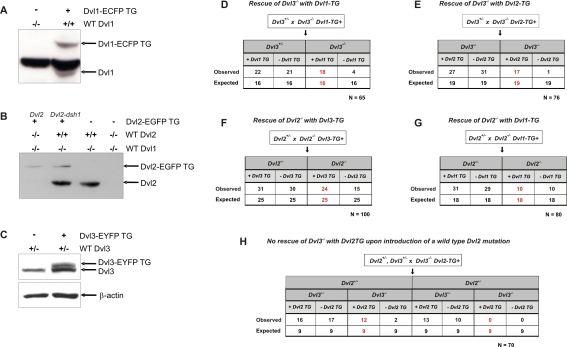
Genetic crosses to demonstrate rescue of *Dvl* mutants with extra copies of other *Dvls*. A genetic approach was used to determine the ability of the *Dvl1-ECFP (Dvl1TG)* and *Dvl2-EGFP* (*Dvl2TG*) BAC transgenes to rescue lethal *Dvl3* ^−/−^ phenotype. Western blot analysis showing relative levels of Dvl1-ECFP (A), Dvl2-EGFP (B) and Dvl3-EYFP (C) compared to protein levels from wild type alleles. Two Dvl2-EGFP transgenes are shown, one representing wild type Dvl2 (left, Dvl2) and one with and a single point mutation that interrupts signaling through the PCP pathway (right, Dvl2-dsh1[38]). *Dvl3* ^−/−^;*Dvl1TG* mutants were crossed with *Dvl3^+/^* ^−^ mice and the progeny genotyped at weaning (D). A similar cross was set up using the *Dvl2TG* and the experiment repeated (E). Parallel crosses using the *Dvl1-ECFP (Dvl1TG)* and *Dvl3-EYFP (Dvl3TG)* were also set up to determine whether additional copies of *Dvl1* or *Dvl3* could rescue *Dvl2* ^−/−^ lethality (F and G, respectively). To confirm that the BAC transgenes were behaving analogous to wild type alleles *Dvl2* ^+/−^;*Dvl3* ^+/−^ mutants were crossed with *Dvl3* ^−/−^;*Dvl2TG* mutants and the surviving progeny were genotyped to test if the *Dvl2TG* could still rescue (H).

The generation of fully functional Dvl transgenes (here and in [6]) allowed us to use a genetic approach to further determine redundancy of function between the Dvl proteins during development. We determined whether an extra copy of either *Dvl1* or *Dvl2*, in the form of *Dvl1-ECFP* and *Dvl2-EGFP* BAC transgenes (which we will refer to here as *Dvl1TG* and *Dvl2TG*, respectively) was able to rescue the lethal *Dvl3* ^−/−^ phenotype. Importantly, western blot analysis revealed that none of the Dvl transgenes were overexpressed compared to protein levels from wild type alleles (Figure 7A–C). As shown above, *Dvl3* ^−/−^ mutants (which still have 2 copies of the *Dvl1* allele and 2 copies of the *Dvl2* allele) cannot survive. Surprisingly, we found that addition of the *Dvl1TG* rescued the *Dvl3* ^−/−^ phenotype such that *Dvl3* ^−/−^;*Dvl1TG* mutants (now *Dvl3* ^−/−^ with 3 copies of *Dvl1* and 2 copies of *Dvl2*) survived. To test whether this was a complete rescue, we crossed these viable *Dvl3* ^−/−^;*Dvl1TG* mutants to *Dvl3* ^+/−^ mice and genotyped the progeny at weaning (Figure 7D). Eighteen *Dvl3* ^−/−^;*Dvl1TG* rescued animals were recovered, compared to 16 that would be expected from normal Mendelian ratios if the *Dvl1TG* is fully able to rescue the *Dvl3* ^−/−^ phenotype. As these crosses were performed in a mixed background, 4 *Dvl3* ^−/−^ mice also survived with out an extra copy of another *Dvl* gene.

Interestingly, in a similar cross using the *Dvl2TG*, we found that the *Dvl3* ^−/−^ lethal phenotype was also rescued by an extra copy of *Dvl2*. We crossed these viable *Dvl3* ^−/−^;*Dvl2TG* mutants with *Dvl3* ^+/−^ mice and genotyped the offspring at weaning (Figure 7E). Seventeen *Dvl3* ^−/−^;*Dvl2TG* mice were recovered, out of 19 expected from a full rescue, indicating approximately 90% rescue of the *Dvl3* ^−/−^ lethal phenotype with additional *Dvl2*.

As 50% of *Dvl2* ^−/−^ mutants die perinatally, we used a similar strategy to determine whether additional copies of *Dvl1* or *Dvl3* could rescue *Dvl2* ^−/−^ lethality. From crosses between *Dvl2* ^−/−^;*Dvl3TG* mutants and *Dvl2* ^+/−^ mice, 24 *Dvl2* ^−/−^;*Dvl3TG* mutants survived, out of 25 expected from a full rescue, indicating approximately 96% rescue of the *Dvl2* ^−/−^ phenotype with an extra copy of *Dvl3* (Figure 7F). However, from crosses between *Dvl2* ^−/−^;*Dvl1TG* mutants and *Dvl2* ^+/−^ mice, only 10 *Dvl2* ^−/−^;*Dvl1TG* mutants were recovered, out of 18 expected from Mendelian ratios if the *Dvl1TG* was able to rescue the *Dvl2* ^−/−^ phenotype. As 50% of *Dvl2* ^−/−^ mutants survive without additional *Dvl* copies, this indicates that adding the *Dvl1TG* was not able to rescue *Dvl2* ^−/−^ mutants (Figure 7G).

To confirm that the BAC transgenes were actually behaving as wild type alleles and that ectopic or inappropriate expression levels were not responsible for rescuing the mutant phenotypes, we genetically reduced the *Dvl* gene dosage by introducing a knock-out *Dvl* allele and again tested the rescue ability of the transgene. *Dvl2* ^+/−^;*Dvl3* ^+/−^ mutants were crossed with *Dvl3* ^−/−^;*Dvl2TG* mutants and the surviving progeny were genotyped (Figure 7H). Twelve *Dvl2* ^+/+^;*Dvl3* ^−/−^;*Dvl2TG* mutants with both wild type alleles of *Dvl2* (a total of 3 copies of *Dvl2*) survived, comparable to 9 expected from normal Mendelian ratios, indicating full rescue. However, no *Dvl2* ^+/−^;*Dvl3* ^−/−^;*Dvl2TG* mutants were recovered, even though 9 were expected from normal Mendelian ratios if the *Dvl2TG* could still rescue. Thus, correction of *Dvl2* dosage from three copies to two copies of *Dvl2* in *Dvl2* ^+/−^;*Dvl3* ^−/−^;*Dvl2TG* mutants (1 wild type allele and the transgene) eliminated the ability of the *Dvl2TG* to rescue the *Dvl3* ^−/−^ mutant phenotype, supporting the conclusion that ectopic expression or over-expression of the transgene was not responsible for the rescue of the *Dvl3* ^−/−^ phenotype.

### Cochlear Defects in *Dvl2*;*Dvl3* Double Mutants

**Figure 8:**
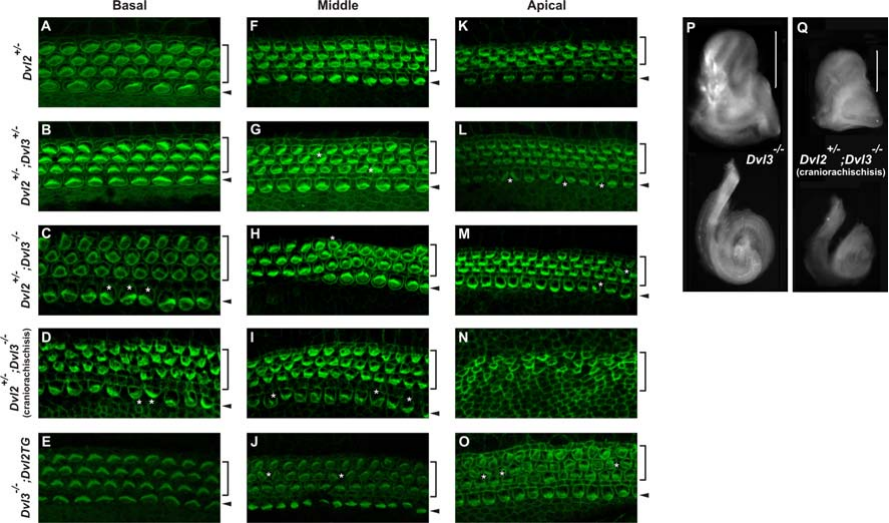
Cochlea defects in double *Dvl2*;*Dvl3* mutants. E18.5 whole mount cochlear confocal images (100×) from *Dvl2* ^+/−^ (A,F,K), *Dvl2* ^+/−^;*Dvl3* ^+/−^ (B,G,L), *Dvl2* ^+/−^;*Dvl3* ^−/−^ (C,H,M), *Dvl2* ^+/−^;*Dvl3* ^−/−^ mutants with craniorachischisis (D,I,N) and *Dvl3* ^−/−^;*Dvl2TG* (E,J,O) mutants. Images show comparable regions in each mutant at the base (A–E), middle (F–J) and apex (K–O) of the cochlear ducts. Brackets and arrowheads indicate the outer and inner hair cells, respectively and white stars indicate misoriented stereociliary bundles. Light micrographs of E18.5 inner ears (top) and isolated cochlea ducts (bottom) from *Dvl3* ^−/−^ mutants (P), and *Dvl2* ^+/−^;*Dvl3* ^−/−^ mutants with craniorachischisis (Q). Brackets indicate the cochlea portion of the inner ear.

Similar cochlear defects have been observed in both *Dvl1* ^−/−^;*Dvl2* ^−/−^ [6] and *Dvl3* ^−/−^ mutants (Figure 4). Therefore, we examined whether all three *Dvls* have redundant functions in the developing organ of Corti. The inner ears of double *Dvl* mutants were studied to determine whether the further loss of an additional *Dvl* gene would worsen the phenotype observed in *Dvl3* ^−/−^ mutants. Only the cochlea from *Dvl1* ^−/−^;*Dvl3* ^+/−^, *Dvl2* ^+/−^;*Dvl3* ^+/−^ and *Dvl2* ^+/−^;*Dvl3* ^−/−^ mutants were examined as other double *Dvl* mutants were lethal too early in development. The organ of Corti from *Dvl1* ^−/−^;*Dvl3* ^+/−^ mutants appeared normal (data not shown), as did those from the *Dvl2* ^+/−^ (Figure 8A,F,K) and *Dvl3* ^+/−^ (not shown) single heterozygote samples. However, several of the sensory hair cells in *Dvl2* ^+/−^;*Dvl3* ^+/−^ double heterozygotes had rotated stereociliary bundles (Figure 8B,G,L). This phenotype appeared worse in the *Dvl2* ^+/−^;*Dvl3* ^−/−^ mutants with the loss of another *Dvl3* allele (Figure 8C,H,M) and mild patterning defects were also observed in one of these mutants (Figure 8H). In the *Dvl2* ^+/−^;*Dvl3* ^−/−^ mutants that displayed craniorachischisis, the phenotype was even more severe (Figure 8D,I,N). Throughout the whole cochlea duct the hair cells did not appear to be fully developed, even in the basal region. Additionally, it is hard to see the typical shape of stereociliary bundles in most of the hair cells and the differentiation of these cells is apparently delayed (Figure 8N). Strong patterning defects were also observed as the cochleae of the *Dvl2* ^+/−^;*Dvl3* ^−/−^ mutants were much shorter compared to *Dvl3* ^−/−^ controls (Figure 8P,Q).

Functional redundancy of the *Dvls* in cochlea development could only be examined in the *Dvl3* ^−/−^ mutants rescued with the *Dvl2-EGFP* transgene. The organ of Corti from *Dvl3* ^−/−^;*Dvl2TG* mutants still displayed misorientation of stereocilia in several of the sensory hair cells (Figure 8E,J,O), similar to the phenotype described for *Dvl3* ^−/−^ mutants (Figure 4), indicating that increasing the copy number of *Dvl2* was not able to restore the correct alignment of stereocilia in the *Dvl3* ^−/−^ mutants.

### Canonical Wnt Signaling in *Dvl* Mutants

**Figure 9:**
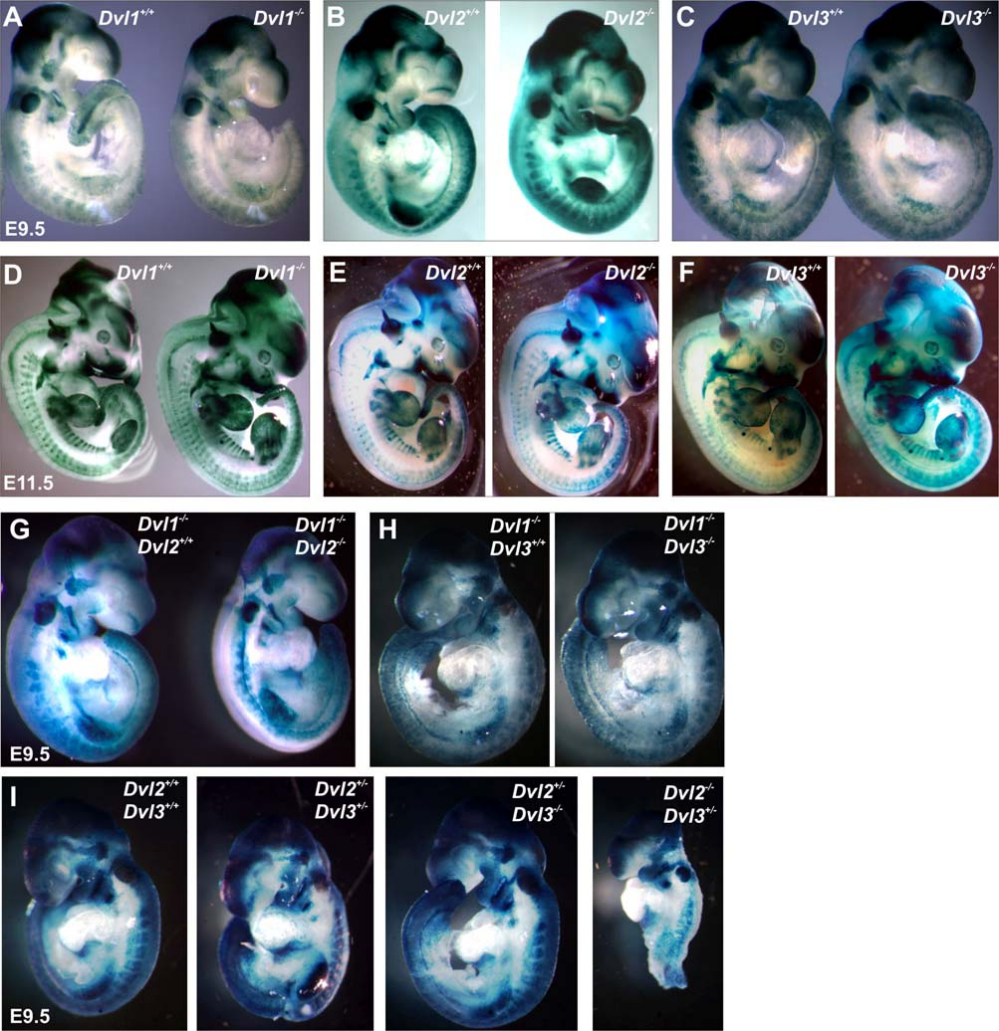
Canonical Wnt signaling in double *Dvl* mutants at mid-gestation. *Dvl* mutants were crossed with BATgal mice that harbor the canonical Wnt LacZ reporter and embryos collected during mid-gestation. Single *Dvl1* ^−/−^ (A,D), *Dvl2* ^−/−^ (B,E) and *Dvl3* ^−/−^ (C,F) mutants carrying the reporter were examined at E9.5 (A–C) and E11.5 (D–F), along with wild-type littermates. Double *Dvl* mutants; *Dvl1* ^−/−^;*Dvl2* ^−/−^ (G), *Dvl1* ^−/−^;*Dvl3* ^−/−^ (H) and *Dv2* ^+/−^;*Dvl3* ^+/−^, *Dvl2* ^+/−^;*Dvl3* ^−/−^ and *Dvl2* ^−/−^;*Dvl3* ^+/−^ (I) with the reporter were isolated at E9.5, with suitable control littermates.

Previous studies from our lab [6],[38] and data presented above are consistent with a role for the Dvls in the PCP pathway during neurulation and cochlear development, while a role for Dvls in Wnt signaling is not as clear. Therefore, BATgal mice, which carry a β-catenin-responsive *LacZ* gene, were used to determine whether canonical Wnt signaling was functional or interrupted in the *Dvl* mutants during mid-gestation. Global canonical Wnt signaling patterns appeared largely unaffected in each of the single *Dvl* mutants compared to their wild type littermates at either E9.5 (Figure 9A–C) or E11.5 (Figure 9D–F). Canonical Wnt signaling was also generally unchanged in the double *Dvl* mutants *Dvl1* ^−/−^;*Dvl2* ^−/−^, *Dvl1* ^−/−^;*Dvl3* ^−/−^, *Dv2* ^+/−^;*Dvl3* ^+/−^, *Dvl2* ^+/−^;*Dvl3* ^−/−^ and *Dvl2* ^−/−^;*Dvl3* ^+/−^ at E9.5 (Figure 9G–I). However, subtle abnormalities were observed. For example, TCF activity appears reduced in the somites of *Dvl1* ^−/−^ mutants (Figure 9A) and in the limb bud of *Dvl2* ^−/−^ mutants (Figure 9B) at E9.5, and the pattern of staining appears altered in both the somites and pharyngeal region of *Dvl2* ^−/−^ mice at E11.5 (Figure 9E).

## Discussion

**Figure 10:**
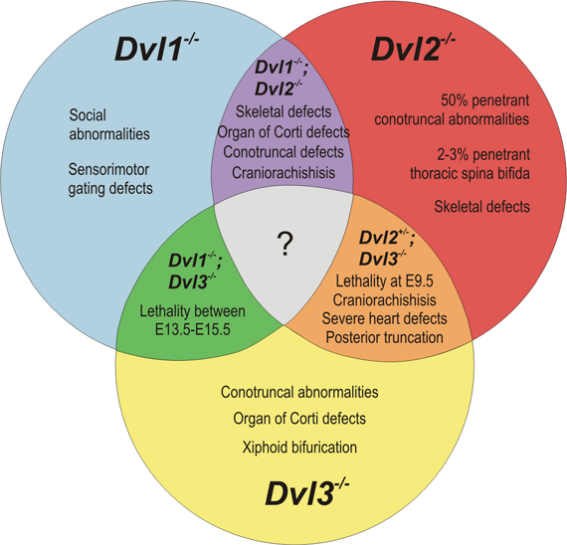
Summary of the phenotypes of single *Dvl* and double *Dvl* mutants. Single *Dvl* mutants display distinct phenotypes, but both novel and more severe phenotypes are observed in the double *Dvl* mutants, indicating functional redundancy between the *Dvl* genes.

A large body of evidence demonstrates that Dvl proteins function in highly conserved pathways in both vertebrates and invertebrates. It is known that in Drosophila [42],[43], Xenopus [37], [44]–[46], mouse [6],[38] and mammalian cell lines [47]–[49], Dishevelled proteins mediate their effects on the canonical and non-canonical Wnt pathways via highly conserved protein domains. As essential components of both the canonical Wnt and PCP pathways, Dvl proteins are required for many developmental processes. A recent publication has addressed the possibility of redundancy between the three Dvl proteins in mammalian cell lines *in vitro* [47]. We further aim to determine the action of the Dvl proteins during development *in vivo*. Having previously described the roles of *Dvl1* and *Dvl2* in mammalian development [38],[40],[41], we now describe the specific developmental roles of *Dvl3* and additionally use well-established genetic approaches to determine the pathways disrupted *in vivo* that account for the phenotypes observed in the *Dvl* mutant mice and to demonstrate redundancy between the *Dvls* during development *in vivo*. A summary of the phenotypes of single *Dvl* and double *Dvl* mutants is shown in Figure 10.

*Dvl3* knockout mice die perinatally with cardiac outflow defects, indicating an important role for Dvl3 in conotruncal development. This is particularly interesting as approximately 30% of all congenital heart diseases are due to defects in this region [50],[51]. The loss of *Dvl3* causes a similar conotruncal phenotype to those observed with loss of *Dvl2* [40], suggesting redundant functions for these homologous genes in outflow tract development. This was supported both by the similar expression patterns of *Dvl2* and *Dvl3* found during conotruncal development and the observation of cardiac abnormalities in double *Dvl2* ^+/−^;*Dvl3* ^+/−^ heterozygotes, as compared to normal hearts of *Dvl2* ^+/−^ and *Dvl3* ^+/−^ single heterozygotes. The increased severity of the cardiac phenotype of the double *Dvl2* ^−/−^;*Dvl3* ^+/−^ and *Dvl2* ^+/−^;*Dvl3* ^−/−^ mutants, compared with the single *Dvl2* ^−/−^ and *Dvl3* ^−/−^ mutants, further indicates redundancy between these *Dvls* in heart development. Finally, with a genetic approach we found that an extra copy of either *Dvl1* or *Dvl2*, using either the *Dvl1-ECFP* or *Dvl2-EGFP* BAC transgene, could rescue lethal *Dvl3* ^−/−^ heart phenotype, demonstrating the ability of both *Dvl1* and *Dvl2* to compensate for the loss of *Dvl3* to enable normal development.

Our data suggests that *Dvl1* shares redundant functions with *Dvl2* and *Dvl3* in cardiac development. However we found that an additional copy of *Dvl1* is not able to rescue the *Dvl2* ^−/−^ cardiac defects. Although the Dvl proteins appear to have the same expression patterns and redundant functions, we have not yet addressed the possibility of differences in protein expression level. There may a certain threshold of Dvl protein required for normal heart development, but under this threshold there is insufficient signaling to permit normal development. Interestingly, a recent paper by Lee et al. demonstrated that in a number of mammalian cell lines all three Dvls are present, but that the relative levels of expression of the three Dvls differ greatly [47].

Normal development of the outflow tract requires the addition of cells from both the CNC and the SHF, and a lack of contribution of either of these tissues is often the cause of defects in the conotruncal region. Further, Dvl2 has previously been found to be important for controlling proliferation of CNC cells, via activation of *Pitx2* [52]. However, both CNC cells and SHF cells appeared to be present in the absence of *Dvl3*, suggesting that the heart defects are not due to a complete lack of either of these tissues. Further studies to determine whether these cells are properly situated in relation to the surrounding tissues, and whether they are indeed still functional, are in progress.

Dvl proteins function in both the canonical and non-canonical PCP pathways, therefore disruption of either or both of these pathways could be the cause of the phenotypes observed in *Dvl3* ^−/−^ mice. Roles for both pathways in heart development have been reported. Canonical Wnt signals appear essential for the proliferation of SHF cells [19]–[23], whereas the PCP pathway is thought to be necessary for the polarized migration of myocardial cells required in the outflow tract septum (Henderson et al., 2006; Phillips et al., 2005). *Dvl3* ^+/−^;*LtapLp*/+ double heterozygotes displayed no abnormal heart phenotype, suggesting that *Dvl3* may function through the canonical and not the PCP pathway during heart development. Conversely, it is possible that the level of PCP signaling was not reduced below the hypothetical threshold required for the phenotype. Further experiments will be required to distinguish these possibilities.

We have shown here that Dvl1 and Dvl3 colocalize in the developing neural tube with similar expression patterns to that of Dvl2 as we described previously [38], and provide further evidence for a similar functional role for *Dvl3* in neurulation as *Dvl1* and *Dvl2* [38]. Although none of the *Dvl* single knockouts display neural tube closure defects, disruption of either *Dvl1* and *Dvl2* or *Dvl2* and *Dvl3* genes (*Dvl1* ^−/−^;*Dvl2* ^−/−^, *Dvl2* ^−/−^;*Dvl3* ^+/−^, *Dvl2* ^+/−^;*Dvl3* ^−/−^) results in incomplete neurulation, suggesting a dosage sensitive redundant role for all three Dvls in this process. Interestingly, *Dvl1* ^−/−^;*Dvl3* ^−/−^ mutants did not display neural tube defects, suggesting that the three *Dvls* are not functionally equivalent. Neural tube defects appear in single *Dvl* mutants when crossed with *LtapLp* mice, (*Dvl2* ^−/−^;*LtapLp*/+ , *Dvl3* ^+/−^;*LtapLp*/+ and *Dvl3* ^−/−^;*LtapLp*/+), indicating genetic interaction with the *Dvls* and the PCP component, *Vangl2* and therefore that the *Dvls* signal through the PCP pathway to promote neural tube closure.

It is interesting that both exencephaly and craniorachischisis are observed in *Dvl3*;*LtapLp* mutants. Craniorachischisis has been observed in many mutants with disrupted PCP signaling, whereas exencephaly is often associated with defective ciliogenesis (reviewed in [53]). Recently a connection between cilia and PCP signaling has been suggested as the ciliary proteins Inversin [54] and Bardet-Biedl Syndrome protein-4 (BBS4) [55] were shown to influence PCP signaling and CE movements. Additionally, disruption of the PCP effectors *inturned* and *fuzzy* in *Xenopus laevis* demonstrated a link between ciliogenesis, PCP signaling and Hedgehog signaling [56]. Localization of both *dishevelled* and *inturned* near the basal apparatus of cilia further suggests a role for PCP components in regulating ciliogenesis [56].

The polarity defects observed in *Dvl3* ^−/−^ cochleae were much more severe with the introduction of an additional single *LtapLp* mutation, indicating that *Dvl3* genetically interacts with *Vangl2* and functions in the PCP pathway to regulate cell polarity in the organ of Corti. The CE movements driven by the PCP pathway that are required for the extension and thinning of the developing organ of Corti were also disrupted in *Dvl3*;*LtapLp* mutants, resulting in shortened organs of Corti relative to controls. Furthermore, the asymmetric localization of Dvl3 during establishment of stereocilia orientation is consistent with the asymmetric localization of Dvl2 [6] and other core PCP components in mammals [34],[57],[58]. The polarized asymmetric arrangement of PCP proteins in the fly wing is also required for the uniform orientation of hairs [59],[60]. The sensory hair cells in this region are surrounded by the cellular processes of various types of supporting cells and tight cell contacts are formed between the cells (reviewed in [32]). Dvl3 appears to be localized on the cellular boundary formed between the sensory hair cells and the projections of the supporting cells, making it difficult to determine whether it is expressed on the lateral side of the hair cell, or on the medial side of the supporting cell. Dvl2 has previously been shown to localize at the lateral side of the hair cell [6] and as our evidence suggests redundant functions for the Dvl proteins, it seems likely that Dvl3 is also expressed here.

Redundancy between the three Dvls in the developing organ of Corti was implied from the similarity of phenotype in *Dvl3* ^−/−^ mutants and *Dvl1* ^−/−^;*Dvl2* ^−/−^ double mutants [6],[38]. Further, a mild PCP phenotype was observed in *Dvl2* ^+/−^;*Dvl3* ^+/−^ double heterozygotes, despite normal development in the single heterozygotes and the defect in *Dvl2* ^+/−^;*Dvl3* ^−/−^ double mutants was much more severe than in the single *Dvl3* ^−/−^ mutant. Interestingly, despite redundancy between the Dvls, addition of the *Dvl2-EGFP* BAC transgene was not able to rescue the rotated stereocilia phenotype in *Dvl3* ^−/−^ mutants. We again propose that a certain threshold of Dvl protein level may be required for normal development and the relative expression levels of the three Dvls in the developing organ of Corti influences the cochlear phenotype and the ability of a *Dvl* transgene to rescue this phenotype.

Interestingly, the *Dvl3* ^+/−^;*LtapLp*/+ and *Dvl3* ^−/−^;*LtapLp*/+ mutants that showed the CE/PCP-phenotype in the neural tube also displayed the PCP-phenotype in the organ of Corti. However, the misorientation phenotype was less severe in both *Dvl3* ^+/−^;*LtapLp*/+ and *Dvl3* ^−/−^;*LtapLp*/+ mutants than previously found in *LtapLp/LtapLp* mutants, in which 95% cells in the two outer hair cell rows are misorientated [6], possibly due to the remaining *Dvl1* and *Dvl2* alleles in these mutants. It was also noted that *Dvl2* ^+/−^;*Dvl3* ^−/−^ mutants with defects in the neural tube had a much more severe phenotype in the organ of Corti than those with normal neurulation, indicating a strong correlation between the PCP-phenotypes in these two tissues.

We have previously shown that both *Dvl1* and *Dvl2* play roles in somite segregation, causing skeletal malformations in mice that lack these genes [40]. Given the high homology between the three *Dvl* genes, we examined the skeletons of *Dvl3* ^−/−^ mice. However, no vertebral or rib malformations were observed. Some mutants did display xiphoid bifurication, although we are unsure of the significance of this phenotype as it was also seen in several wild type controls. Severe skeletal defects involving truncation of the A–P axis were, however, observed in *Dvl2* ^−/−^;*Dvl3* ^+/−^ double mutants, supporting previous evidence to suggest redundant roles of the *Dvls* in somite formation. A similar phenotype of lack of caudal somites and absence of tail bud formation is seen in mice homozygous for a null *Wnt3a* allele (*Wnt3a^neo^*) [61], and a less severe phenotype appears in the hypomorphic *Wnt3a* allele mutant, *vestigial tail*, which shows loss of caudal vertebrae causing a shortening of the tail [62]. Mice lacking *Wnt5a* are also shortened along the A–P axis with a phenotype similar to *Dvl2* ^+/−^;*Dvl3* ^−/−^ mutants, and share outgrowth defects in the developing face and lack of tail [63]. Wnt3a is classically considered to stimulate canonical Wnt signals, whereas Wnt5a is normally associated with non-canonical Wnt mechanisms, indicating that further investigation is needed to determine which pathway or pathways Dvl is required to signal through for the normal development of these structures. We grossly examined Wnt signaling in both single and double *Dvl* mutants using the TOPflash reporter of TCF activity. Interestingly we found that global Wnt signaling was largely unaffected in even the most severely affected mutants, suggesting that only a low level of Dvl is sufficient for functional canonical Wnt signals. However, our data also suggests subtle abnormalities in Wnt signaling. Precise determination of these abnormalities will require careful sectioning of specific tissues and examination of various cell types throughout development.

The three highly homologous Dvl proteins shared in mammals have very similar broad expression patterns in development. This study completes the initial characterization of the specific, individual roles of each of these proteins and also establishes functional redundancy and overlap in a number of developmental processes. Dvl3 is required for the development of the cardiac outflow tract and signals in the PCP pathway to regulate CE in the developing neural tube and cochlea, as well as cell polarity in the organ of Corti. Dvl1 and Dvl2 are redundant with each other [6],[38],[40] as well as with Dvl3 in a number of these developmental processes.

## Materials and Methods

### Mouse Strains and Animal Care

All animal care and experiments were performed under protocols approved by the NHGRI/NIH and UCSD Animal Care and Use Committees. *LtapLp* mutants were originally acquired from Jackson Laboratory, *Wnt1-Cre* mice were a kind gift of Dr. Andrew McMahon, Harvard and *Isl1-Cre* mice were a generous gift from Sylvia Evans, UCSD. We generated the *Dvl3* ^−/−^ and *Dvl3-EYFP* mouse mutants as described in Figures S1 and S2, respectively. Genotyping of the *Dvl3* mutants was performed with the following primers, *Dvl3* forward 5′-TCCGATGAGGATGATTCCACC-3′, *Dvl3* reverse 5′-TGAGGCACTGCTCTGTTCTGT -3′, *Dvl3* knockout 5′-TTGGCCCACAATGGAGATGCCC-3′, NLpgk *neo* forward 5′-AGGCTTACCCGCTTCCATTGCTCA-3′. The PCR primers used to distinguish between the transgene and wild type *Dvl3* allele were Dvl3lnt2 forward 5′-GGACGCAGGAGATCTTTGAA-3′ and Dvl3Int2 reverse 5′-CATAGCTGGGGTTGAAGCTC-3′ which amplify a band of 155 bp for the wild type allele and 189 bp for the transgene, due to the presence of the LoxP site. Genotyping for the following mouse mutants have previously been described; *Dvl1* [41], *Dvl2* [40], *Dvl2-EYFP* transgene [6], *Wnt1-Cre* [64], *Isl1-Cre* [18], *Rosa-26-lacZ Cre* [65] and *BAT-gal* [66].

### Scanning Electron Microscopy

After fixing in 3% aldehyde solution (1.5% paraformaldehyde, 1.5% glutaraldehyde) in 0.1 M phosphate buffer pH 7.5, E18.5 hearts were stored in 100% ethanol following dehydration through a graded ethanol series. Hearts were critically point dried, mounted and coated with 300 Angstrom gold-palladium. A Cambridge Instrument Stereoscan 360 scanning electron microscope (Scripps Institute of Oceanography Analytical Facility) was used to view the prepared samples.

### Histology

P0 hearts were dissected into 10% buffered formalin, dehydrated, embedded in paraffin wax, sectioned at 8 µm thickness and stained with hematoxylin and eosin using standard methods.

To label the CNC and SHF cell populations in *Dvl3* ^−/−^ hearts, *Dvl3* ^+/−^ mice carrying *Wnt1-Cre* [64] and *Isl1-Cre* [18], respectively, were crossed with *Dvl3* ^+/−^ mice carrying the *Rosa-26-lacZ Cre* reporter gene [65]. Hearts dissected from embryos collected at E14.5 and E18.5 were stored in 30% sucrose in PBS at 4°C overnight, and then embedded in 7.5% gelatin, 15% sucrose. Sections (20 µm) were cut at −24°C on a cryostat and fixed in 2% formaldehyde, 0.2% glutaraldehyde, 0.02% NP-40 and 0.01% sodium deoxycholate. After washing, the slides were stained with 1 mg/ml X-gal in 35 mM potassium ferrocyanide, 35 mM potassium ferricyanide, 2 mM MgCl_2_, 0.02% NP-40, 0.01% sodium deoxycholate at 37°C overnight. After re-fixing in 4% paraformaldehyde, the slides were counterstained with Nuclear Fast Red (Vector Lab), according to the manufacturer's protocol. Whole embryos from the above crosses were also collected at E10.5 and stained for β-galactosidase activity using the same procedures. All images were captured using a Spot 2 camera mounted on a Leica DMR light microscope.

To visualize canonical Wnt signaling, *Dvl* mutant mice were crossed with mice carrying the *BAT-gal* reporter gene [66] and embryos collected at various stages of development. Staining for β-galactosidase activity was performed as described above.

### Confocal Microscopy

To visualize Dvl3 expression, *Dvl3-EYFP* embryos or cochleae were fixed in 4% paraformaldehyde for 30 minutes or overnight at 4°C. To examine the stereocilia in the sensory hair cells, organs of Corti were stained with fluorescein- rhodamine-conjugated phalloidin (Molecular Probes). Both native YFP signal and fluorescent staining was observed using an Olympus FV 1000 or a Zeiss LSM 510 confocal scanning microscope.

### Patterning Defect Quantification in the Cochlea

To measure the severity of the patterning defect, the whole cochlea duct was scanned and the cells numbered for length reference. The number of cells in the defective regions (where there were no longer three outer hair cell rows) was then calculated as a percentage of total cells.

## Supporting Information

Figure S1 Targeted disruption and generation of *Dvl3* deficient mice. A *Dvl3* genomic clone was isolated from a 129 genomic DNA library in FIX II (Stratagene) as previously described [13]. *Dvl3* genomic fragments were subcloned into pBluescript KS II (Stratagene) to enable efficient generation of a *Dvl3* knock-out construct (A). A 3.6 kb BamH-Bgl2 fragment from within intron 1 to the middle of exon 2 and a 2.5 kb Dra1-Not1 (from vector MCS) fragment from within intron 7 to beyond 13 were cloned into pPNT [67] either side of the neomycin resistance gene and in the opposite direction to this marker (A). Gene targeting of this construct in TC1 embryonic stem (ES) cells [68] was used to generate the knockout mice and individual clones were selected and screened by Dra1 digest of genomic DNA with the 3′ probe. Correct targeting of clone 72 (1 of 100 clones) resulted in the presence of a 7.8 Kb targeted allele and a 6 Kb wildtype allele (B). To confirm homologous recombination, a 5′ probe was used to detect a 5 Kb fragment in Nco1 digested ES clone genomic DNA (B). This correctly targeted clone was injected and successfully transmitted through germline. Lines were established in both mixed (129S6×NIH Black Swiss) and uniform (129S6) genetic backgrounds, as described [68]. F_2_ litters contained wild type and heterozygous offspring. *Dvl3^−/−^* embryos were collected from crosses between heterozygotes and western blot analysis using E13.5 whole embryo lysates confirmed the absence of Dvl3 protein (C). PCR primers (listed in methods) were designed to distinguish wild type, heterozygous and homozygous genotypes (D).
(0.5 MB TIF)

Figure S2 Generation of EYFP-tagged *Dvl3* transgene. An EYFP-tagged *Dvl3* transgene was generated using homologous recombination of BACs (A), as described previously [38]. Briefly, BAC clones containing the whole *Dvl3* genomic region, including flanking sequences, were identified using overlapping PCR primers covering the entire region from a BAC library (Genome Systems). BAC modifications were performed as previously described [69], using a SacB-Neo selection cassette (A). An EYFP cassette was fused in-frame to the last codon of *Dvl3* and LoxP sites were introduced within intron 2 and the 3′ UTR flanking region. Western blot analysis of transgenic mouse E13.5 embryo lysates was used to confirm expression of the transgene, which was larger than the wild type Dvl3 protein due to the additional EYFP (3B). PCR primers (listed in methods) binding either side of the LoxP site in intron2 (red arrows in A) were used to distinguish between the transgene and wild type *Dvl3* allele (C).
(3.8 MB TIF)
