# Supplementary figures and images for: Murine Dishevelled 3 Functions in Redundant Pathways with Dishevelled 1 and 2 in Normal Cardiac Outflow Tract, Cochlea, and Neural Tube Development
Source: PLoS Genet. 2008 Nov 14;4(11):e1000259. doi: 10.1371/journal.pgen.1000259 (PMC2576453; doi:10.1371/journal.pgen.1000259)

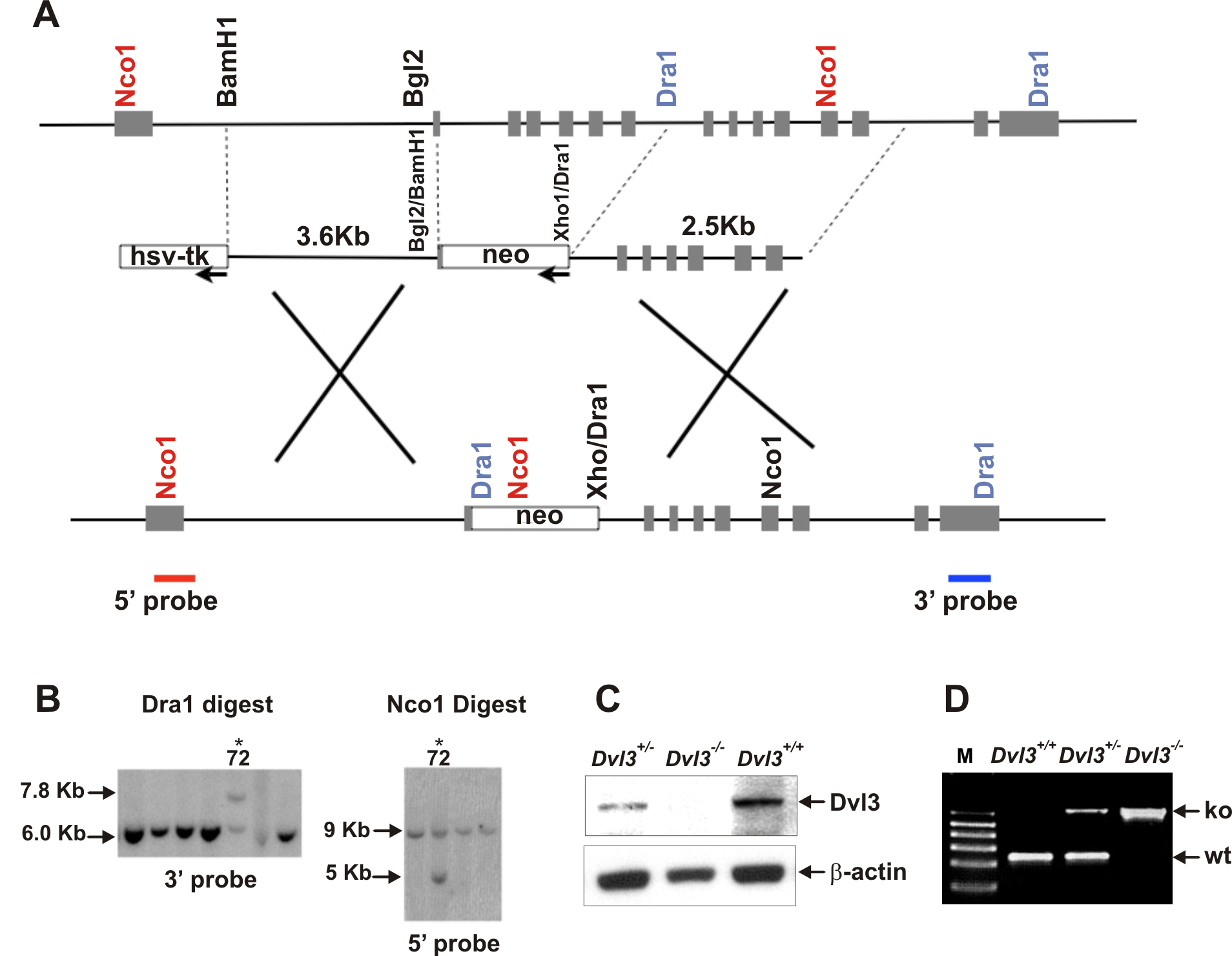

Supplement: Figure S1 — Targeted disruption and generation of Dvl3 deficient mice. A Dvl3 genomic clone was isolated from a 129 genomic DNA library in FIX II (Stratagene) as previously described [13]. Dvl3 genomic fragments were subcloned into pBluescript KS II (Stratagene) to enable efficient generation of a Dvl3 knock-out construct (A). A 3.6 kb BamH-Bgl2 fragment from within intron 1 to the middle of exon 2 and a 2.5 kb Dra1-Not1 (from vector MCS) fragment from within intron 7 to beyond 13 were cloned into pPNT [67] either side of the neomycin resistance gene and in the opposite direction to this marker (A). Gene targeting of this construct in TC1 embryonic stem (ES) cells [68] was used to generate the knockout mice and individual clones were selected and screened by Dra1 digest of genomic DNA with the 3′ probe. Correct targeting of clone 72 (1 of 100 clones) resulted in the presence of a 7.8 Kb targeted allele and a 6 Kb wildtype allele (B). To confirm homologous recombination, a 5′ probe was used to detect a 5 Kb fragment in Nco1 digested ES clone genomic DNA (B). This correctly targeted clone was injected and successfully transmitted through germline. Lines were established in both mixed (129S6×NIH Black Swiss) and uniform (129S6) genetic backgrounds, as described [68]. F2 litters contained wild type and heterozygous offspring. Dvl3−/− embryos were collected from crosses between heterozygotes and western blot analysis using E13.5 whole embryo lysates confirmed the absence of Dvl3 protein (C). PCR primers (listed in methods) were designed to distinguish wild type, heterozygous and homozygous genotypes (D). (0.5 MB TIF) [file pgen.1000259.s001.tif]

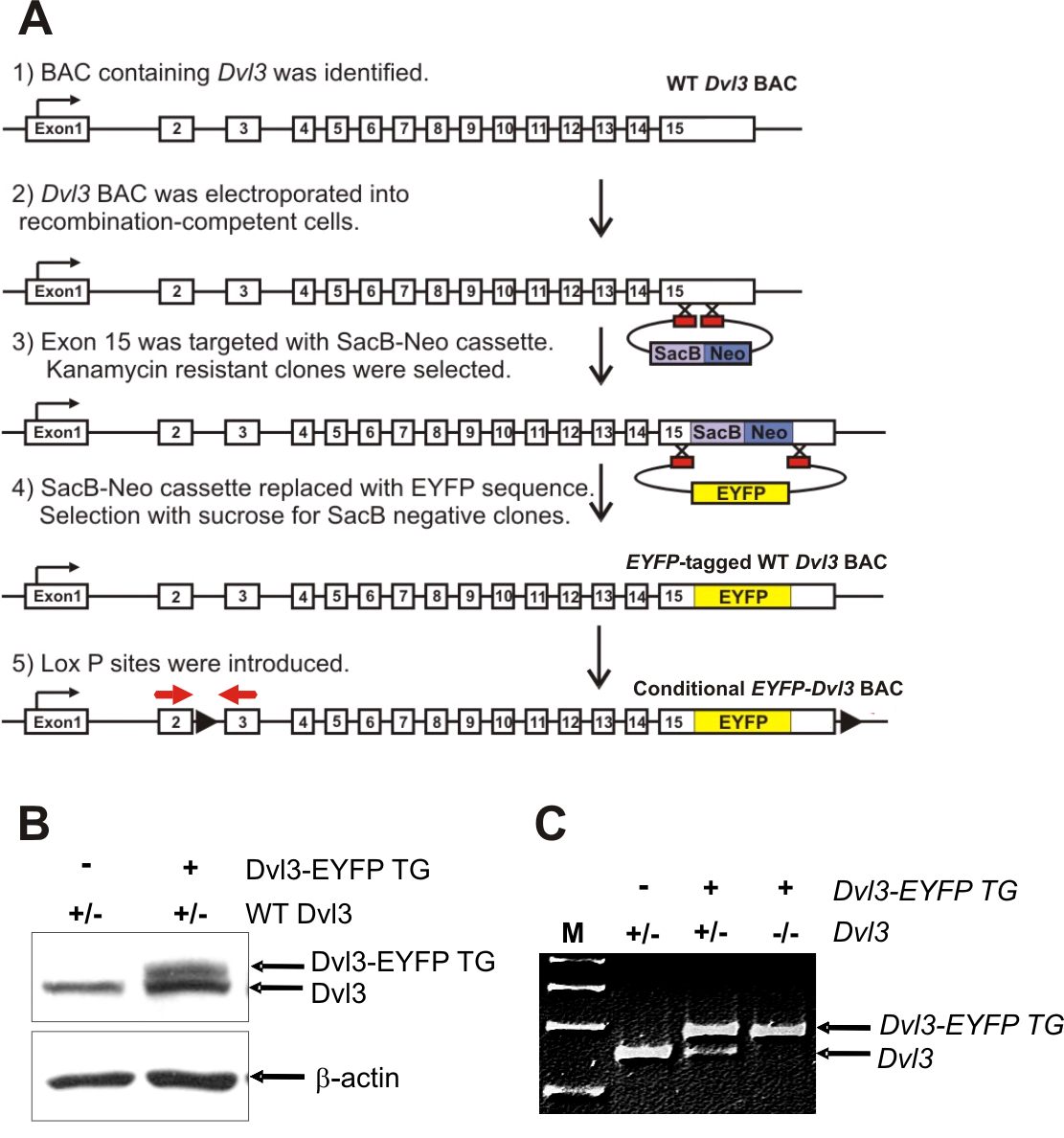

Supplement: Figure S2 — Generation of EYFP-tagged Dvl3 transgene. An EYFP-tagged Dvl3 transgene was generated using homologous recombination of BACs (A), as described previously [38]. Briefly, BAC clones containing the whole Dvl3 genomic region, including flanking sequences, were identified using overlapping PCR primers covering the entire region from a BAC library (Genome Systems). BAC modifications were performed as previously described [69], using a SacB-Neo selection cassette (A). An EYFP cassette was fused in-frame to the last codon of Dvl3 and LoxP sites were introduced within intron 2 and the 3′ UTR flanking region. Western blot analysis of transgenic mouse E13.5 embryo lysates was used to confirm expression of the transgene, which was larger than the wild type Dvl3 protein due to the additional EYFP (3B). PCR primers (listed in methods) binding either side of the LoxP site in intron2 (red arrows in A) were used to distinguish between the transgene and wild type Dvl3 allele (C). (3.8 MB TIF) [file pgen.1000259.s002.tif]
